# 18F-FDG PET/CT in a Patient With Malignant Pheochromocytoma Recurrence and Bone Metastasis After Operation—Case Report and Review of the Literature

**DOI:** 10.3389/fmed.2021.733553

**Published:** 2021-11-12

**Authors:** Bei Feng, Maojia Chen, Yanghongyan Jiang, Yongfeng Hui, Qian Zhao

**Affiliations:** ^1^Department of Nuclear Medicine, General Hospital of Ningxia Medical University, Yinchuan, China; ^2^West China Hospital of Sichuan University, Chengdu, China

**Keywords:** 18F-FDG, malignant pheochromocytoma, brown fat, recurrence, bone metastasis

## Abstract

**Introduction:** Bone metastasis of malignant pheochromocytoma is a rare disease. We report a patient with a 10-year history who underwent 18F-FDG PET/CT to detect bone metastasis and receive radiotherapy and chemotherapy with complete response for bilateral iliac pain.

**Case presentation:** A 48-year-old male patient complained of dizziness, hypertension, and bilateral iliac pain for 2 months. The patient had a history of resection of bilateral malignant adrenal pheochromocytoma 10 years earlier, and all complaints were relieved immediately after operation. 18F-FDGPET/CT showed abdominal lymph node uptake and multiple bone uptake, as well as multiple brown fat uptake. A biopsy of the left ilium confirms the metastasis of malignant pheochromocytoma.

**Discussion:** In our literature review, we discuss the metastasis of pheochromocytoma reported by some scholars, and the role of radionuclides such as 18F-FDG PET/CT, 18F-DOPA PET/CT, I-123MIBG, and 68Ga-DOTATATE PET, in the diagnosis of malignant pheochromocytoma. The patient above is a good case for clinicians in the diagnosis and treatment of metastatic pheochromocytoma, especially in some hospitals with only 18F-FDG imaging agents.

**Conclusion:** A review of this case and similar rare cases in the literature illustrates the importance of 18F-FDG PET/CT in the diagnosis of malignant pheochromocytoma.

## Introduction

According to the classification of tumors of the World Health Organization, 3–13% of patients with adrenal pheochromocytoma are diagnosed as malignant lesions (1). The 5-year survival rate was 34–60%, depending on the site of tumor metastasis. Patients with liver and lung metastasis were shown shorten survival time, while those with only bone metastasis showed the best prognosis (2). Unfortunately, it is extremely rare that the pheochromocytoma metastasizes to the bone.

Currently, the commonly used imaging for detecting bone metastasis is CT, MRI, PET/CT, and SPECT/CT. Among them, PET/CT or SPECT/CT is a “one-stop” examination, which displays not only morphological but also metabolic information of the whole body by one test. 18F-FDG is the analog of glucose, which reflects the metabolism of glycolysis inside the tumors. 18F-FDG PET/CT has been widely used in the detection, staging, prognosis, and therapeutic assessment of various kinds of tumors. Besides the primary lesions, all metastatic lesions can be shown, including osteogenic and osteolytic bone destructions (3). Pacak et al. (4) found that 18F-FDG PET/CT imaging is helpful for the localization and diagnosis of pheochromocytoma with high sensitivity and specificity.

Here, we report a rare case of 10-year postoperative bone metastasis of malignant pheochromocytoma detected by 18F-FDG PET/CT. The patient has provided a written informed consent form and agreed to publish this manuscript and any identifiable images or data.

## Case Presentation

We report a 48-year-old male patient with dizziness, hypertension, bilateral iliac pain, and elevated blood pressure for 2 months. Bilateral adrenalectomy and splenectomy were performed in 2009. Postoperative pathology confirmed bilateral adrenal pheochromocytoma. All the symptoms were completely relieved, and the blood pressure returns to a normal level after operation. No antitumor and antihypertension therapy were needed. In April 2020, the patient visited the hospital and complained bone pain in the left hip, accompanied by palpitation, shortness of breath, and dizziness. The highest blood pressure was 200/120 mmHg, cortisol 4PM was 6.54 μg/dl (3.44–16.76), cortisol 8PM was 13.64 μg/dl (5.27–22.45), and serum corticotropin was 167.9 pg/ml (7.20–63.30). Chest X-ray showed no abnormality, UCG: left ventricular diastolic function decreased (Grade I), aortic sinus widened. T3, T4, TSH, CEA, CA125, CA199, PSA, HCG, NSE, CY-FRA21-1, and other tumor-related markers were normal. Urapidil sustained-release tablets were not effective, and the blood pressure control was not satisfied. The bone pain was obviously aggravated in nearly half a month.

In order to further find out the cause of bone pain, the patient underwent 18F-FDG PET/CT examination in our hospital. After fasting for more than 4 h, 7.91 mCi was injected intravenously, and 18F-FDG PET/CT brain body tomography was performed after 60-min rest.

**Figure 1:**
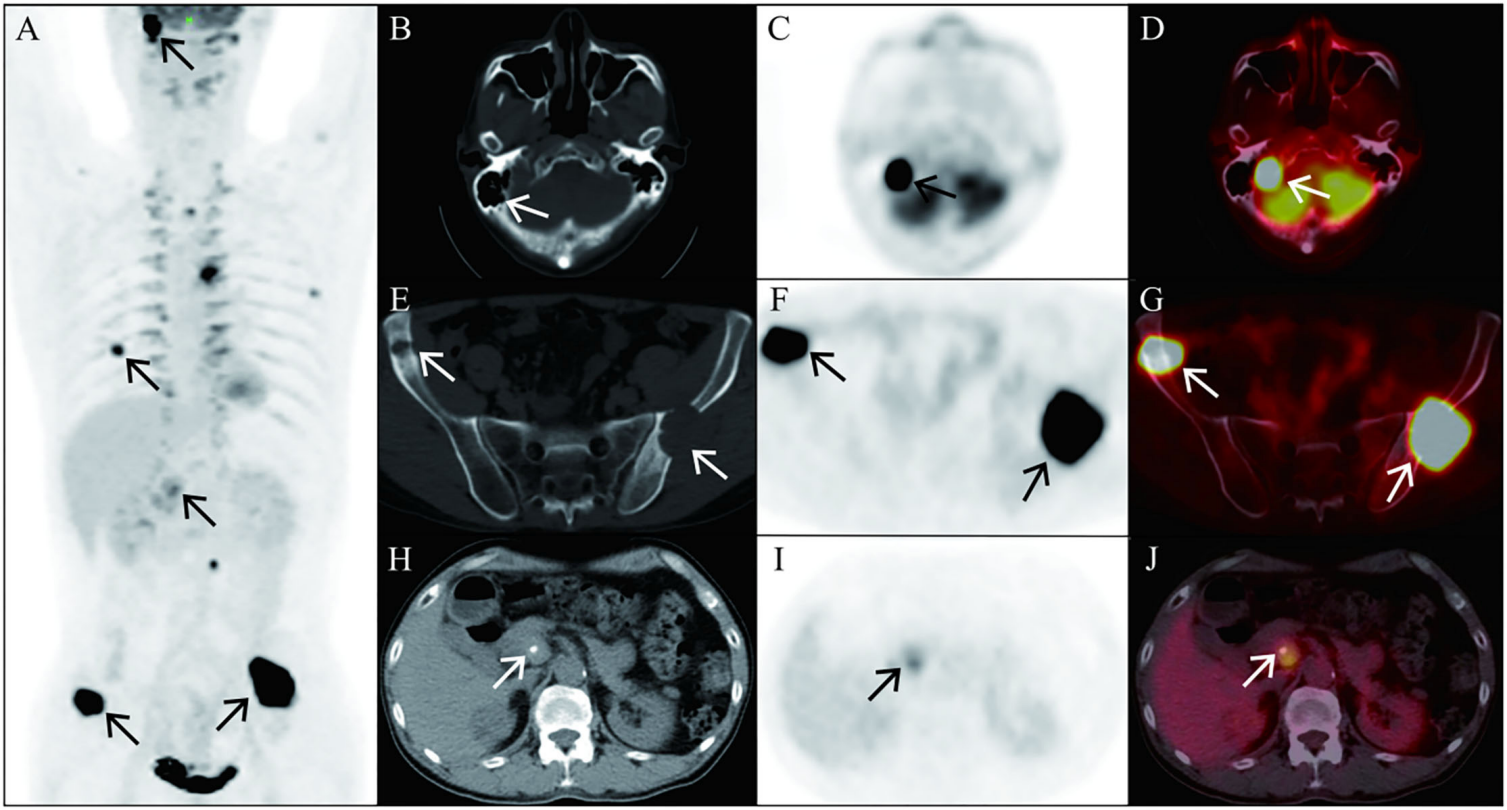
As shown in MIP of 18F-FDG PET, 18F-FDG uptake in the petrous part of right temporal bone [**(A)**, arrows], right foramen magnum, right four anterior ribs, left five lateral ribs and posterior ribs, left eight posterior ribs [**(A)**, arrows], left pedicle of Lumbar 3 vertebral body [**(A)**, arrows], and bilateral ilium [**(A)**, arrowhead] are revealed and characterized with osteolytic bone destruction. Axial CT, PET, and fusion PET/CT images of representative involve bone lesions, including the petrous part of right temporal bone [**(B–D)**, arrows; SUVmax, 20.3] and bilateral ilium [**(E–G)**, arrowhead; SUVmax, 20.3], which are exhibited. In addition, the image shows the 18F-FDG uptake in the abdominal lymph nodes peri-pancreatic, indicating pheochromocytoma metastasis [**(H–J)**, arrow; SUVmax, 5.2].

**Figure 2:**
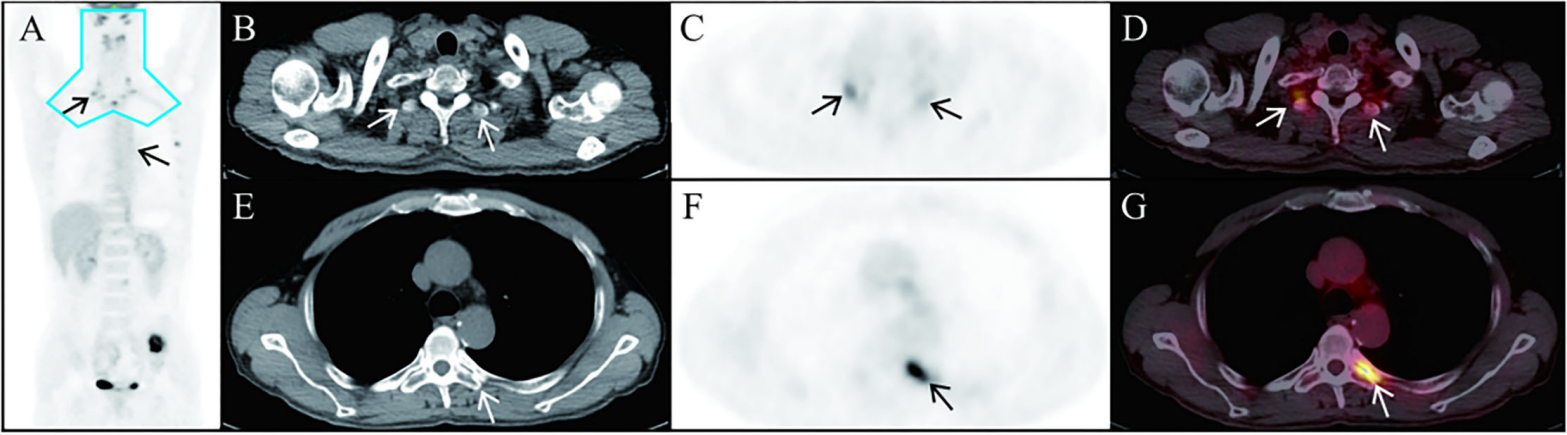
In this case, 18F-FDG uptake (SUVmax, 6.5) is seen in the bilateral vertebrae and intercostal muscles. Representative brown adipose tissue in the thoracic vertebra transverse process [**(B–D)**, arrows] and in posterior intercostal space [**(E–G)**, arrowhead] in the axial CT, PET, and fusion PET/CT images is exhibited. The most common location for brown adipose tissue that is detectable in adults by PET/CT is the blue area in **(A)**.

**Figure 3:**
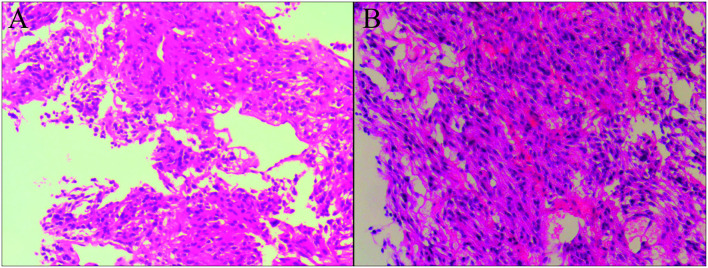
Biopsies of the left ilium demonstrated the presence of metastasis of malignant pheochromocytoma. That tumor cells are spindle shaped and oval, with abundant cytoplasm and pink staining, and the nuclei are round, oval, and deeply stained, which are observed in the microscopic section of hematoxylin-eosin stain [**(A,B)**, original magnification × 10].

An 18F-FDG PET/CT scan reported abdominal lymph node metastasis (SUVmax, 5.2) and multiple bone metastasis (SUVmax, 20.3) (Figure 1), accompanied by FDG uptake of bilateral vertebrae and intercostal muscles (SUVmax, 6.5) related to brown fat uptake (Figure 2). The bone biopsy of left iliac (Figure 3) considered metastasis of malignant pheochromocytoma. Combined with medical history of bilateral adrenal pheochromocytoma and immunohistochemical results: Cha (-), Vim (+ +), AE1/AE3 (-), the case was finally confirmed malignant pheochromocytoma metastasis.

The patients received cyclophosphamide, vincristine, and dacarbazine (CVD) chemotherapy for three cycles. Then, bilateral iliac metastatic lesions were treated with high-dose-rate interstitial brachytherapy (54Gy/3Gy/18f). Finally, the pain in the ilium of the patient was completely relieved after radiotherapy and chemotherapy.

## Discussion

Pheochromocytoma is a benign tumor derived from chromaffin tissue adjacent to adrenal medulla or sympathetic ganglion (5). About 10% of the tumors are bilateral, 10% are non-functional, and 10% are malignant (6). The metastasis has occurred when the malignant pheochromocytoma was found. The only diagnostic criterion for malignant pheochromocytoma is the detection of tumor metastasis (7). Metastases can be isolated or multifocal, commonly in bone, and rarely metastatic to lymph, lung, and liver. The order of involvement was long bone, pelvis, skull, vertebral body and ribs, mostly osteolytic destruction (8).

Studies show that the 5-year survival rate of patients with pheochromocytoma without metastasis can reach 89.3% (9). However, the prognosis of patients with distant metastasis is very poor, with a 5-year survival rate of about 40% and a low long-term survival rate (10). Press et al. (11) believed that recurrence was difficult to treat, especially if diagnosis was not in time or disease had metastatic spread. Therefore, early diagnosis of patients with pheochromocytoma, with malignant potential and careful evaluation of the location and extent of the tumor, is of great significance for the cure of the tumor and the prolongation of survival of the patients.

We searched PubMed with the words “pheochromocytoma” and “osseousmetastasis,” and a total of 35 cases have been reported. McCarthy et al. (12) reported a case of pheochromocytoma metastatic to the left femur, which was detected by X-ray in 1977. They suggested surgical treatment was a nice method for patients with isolated bone metastasis. Kasliwal et al. (13) reported a case of metastatic malignant pheochromocytoma in vertebrae with MRI SPECT imaging and 131I-MIBG therapy. Darlong et al. (14) also reported a bladder and vertebral and rib metastasis detected by 18F-DOPA PET.

In 18F-FDG PET/CT scans, the incidence of brown adipose tissue uptake is 1.44–6.7% for physiological reasons (15). The most common location of brown adipose tissue detected by adult 18F-FDG PET/CT is the blue area in Figure 2A. In this case, there is brown fat uptake in the intercostal muscle, suggesting a recurrence of pheochromocytoma. On the other hand, in recent years, 18F-FDGPET/CT has confirmed the existence of metabolically active brown fat under pathological conditions (16). The increase of brown adipose tissue in patients with pheochromocytoma is associated with high levels of serum catecholamine secretion (17).

More and more new molecular probes or agents were introduced to modality imaging techniques, especially PET/CT. Besides FDG, there are many new probes in radiopharmaceuticals were reported, which played an important and promising role in the diagnosis and therapeutic assessment of metastatic malignant pheochromocytoma.

A SPECT radionuclide tracer, 123I-MIBG, has high specificity in the localization and diagnosis of pheochromocytoma, and is of great significance in ectopic pheochromocytoma, postoperative reexamination, and multiple diseases (18). In addition, Grassi et al. (19) reported a patient with malignant pheochromocytoma who underwent 18F-DOPA PET/CT, I-123MIBG, and 68Ga-DOTATATE PET examinations. The results show that I-123MIBG imaging has traditionally been used to detect pheochromocytoma, but recent studies have shown that some pheochromocytoma imaging may be false negative. Therefore, in order to better display pheochromocytoma, we can consider using new imaging agents, such as 18F-DOPA PET/CT and 68Ga-DOTATATE PET.

In addition, Yanagi et al. (20) reported a primary lesion of the right adrenal, illustrating low uptake due to wide centric necrosis, and metastatic lesions of liver, lumber vertebrae, ribs, and sacroiliac joint showed high uptake on the I-131 MIBG scintigraphy. 131I-MIBG can be selectively absorbed by pheochromocytoma and stored in catecholamine granules of tumor cells to release β-rays in particles and act on tumor cells to achieve therapeutic effect. 131I-MIBG palliative treatment has a certain effect (21). A recent study using FDG-PET, 18F-fluorodopamine PET, MIBG, 111In-pentetreotode, and 99mTc-methylene diphosphate scintigraphy indicated that FDG-PET was the preferable functional imaging modality for the localization of metastatic paraganglioma (22). Jaiswal et al. (23) studies show that 177Lu-DOTATATE is a safe and effective method for the treatment of metastatic pheochromocytoma. Win et al. (24) retrospectively reviewed five patients with malignant phaeochromocytoma who underwent imaging with CT and 123I-MIBG and compared the results with those of PET imaging using 68Ga-DOTATATE. The results showed that, compared with 123I-MIBG, 68Ga-DOTATATE showed more lesions, a higher uptake rate, and better resolution.

The patient above is a good case for clinicians in the assessment of metastatic pheochromocytoma, especially in some hospitals with only 18F-FDG imaging agents.

## Concluding Remarks

Bone metastasis of malignant pheochromocytoma is a rare disease with poor prognosis. For patients with the potential for malignant recurrence, it is necessary to screen valuable predictors of recurrence, strengthen follow-up, and find recurrent diseases. This will help to completely cure and improve the prognosis of patients. Our case shows that 18F-FDG PET/CT has a good value in the localization diagnosis and clinical staging of malignant chromaffin bone metastasis. In addition, brown fat imaging can evaluate the level of catecholamine secretion in diseased blood and very helpful for clinical diagnosis of recurrent malignant pheochromocytoma.

## Data Availability Statement

The original contributions presented in the study are included in the article/supplementary material, further inquiries can be directed to the corresponding author/s.

## Ethics Statement

Ethical review and approval was not required for the study on human participants in accordance with the local legislation and institutional requirements. The patients/participants provided their written informed consent to participate in this study. Written informed consent was obtained from the individual(s) for the publication of any potentially identifiable images or data included in this article.

## Author Contributions

BF and MC initiated the idea for case reporting and prepared the final copy of the manuscript. YH and YJ took responsibility for collecting patient's data. QZ took responsibility for reviewing the CT and FDG PET/CT. All authors have read and approved the final manuscript.

## Conflict of Interest

The authors declare that the research was conducted in the absence of any commercial or financial relationships that could be construed as a potential conflict of interest.

## Publisher's Note

All claims expressed in this article are solely those of the authors and do not necessarily represent those of their affiliated organizations, or those of the publisher, the editors and the reviewers. Any product that may be evaluated in this article, or claim that may be made by its manufacturer, is not guaranteed or endorsed by the publisher.
